# An anesthetic perspective on transoperative complications in open versus robot-assisted radical cystectomy: a five-year retrospective study

**DOI:** 10.1016/j.bjane.2025.844674

**Published:** 2025-08-28

**Authors:** Sérgio Luiz do Logar Mattos, Ronaldo Damião, Fabrício Borges Carrerette, Aretha Paes de Lima Carneiro, Ian Maia Fontes

**Affiliations:** a Universidade do Estado do Rio de Janeiro (UERJ), Hospital Universitário Pedro Ernesto, Rio de Janeiro, RJ, Brazil; b Universidade do Estado do Rio de Janeiro (UERJ), Faculdade de Ciências Médicas, Rio de Janeiro, RJ, Brazil

**Keywords:** Anesthesia, Cystectomy, Death, Robot surgery

## Abstract

**Background:**

Radical cystectomy remains the standard treatment for invasive bladder cancer, yet it carries significant anesthetic risks. While robot-assisted surgery has gained popularity, data comparing its anesthetic implications to those of open surgery are limited. This study aimed to compare the incidence of transoperative complications between the two techniques.

**Methods:**

We retrospectively analyzed 44 patients who underwent open (n = 29) or robot-assisted (n = 15) radical cystectomy in a university hospital between 2019 and 2024. Data were collected on American Society of Anesthesiologists (ASA) physical status, intraoperative hemodynamic parameters, ventilatory complications, additional postoperative opioid requirements, Intensive Care Unit (ICU) stay, and total length of hospital stay. Correlations between blood loss, transfusion requirements, and hemodynamic variables were evaluated.

**Results:**

The robotic cystectomy group experienced less intraoperative bleeding (mean of 410 ± 185 mL vs. 662.5 ± 210 mL; p = 0.002), but no significant reduction in transfusion requirements (95% CI not reported; p = 0.110) despite a strong correlation between bleeding volume and need for transfusion (*r* = 0.78; p < 0.001). Opioid consumption was significantly higher in the open cystectomy group (75.9% vs. 33.3%; p = 0.004). There was no significant difference in intraoperative hypotension, vasoactive drug use, ventilatory complications, in-hospital mortality, ICU stay, or total hospital stay (p > 0.05 for all). However, the small sample size limits the precision of these estimates.

**Conclusion:**

While robot-assisted radical cystectomy was associated with reduced blood loss and lower additional postoperative opioid use, our small retrospective sample did not identify significant differences in intraoperative hemodynamic parameters or major complications. The surgical technique had no impact on in-hospital mortality.

## Introduction

The increasing life expectancy, especially in low- and middle-income countries, has contributed to a rise in bladder cancer incidence. In Brazil alone, an estimated 11,370 new cases are expected annually between 2023 and 2025.^1^ Radical cystectomy remains the gold standard treatment for localized muscle-invasive tumors and non-muscle-invasive disease with a high risk of recurrence. While effective, this procedure carries significant morbidity and mortality risks.

Recent years have seen the emergence of Robot-Assisted Radical Cystectomy (RARC) as a minimally invasive alternative, associated with less intraoperative bleeding, faster recovery, and shorter hospital stay.^2^ However, studies such as the RAZOR trial and Cochrane meta-analyses suggest that, despite these perioperative benefits, RARC does not significantly differ from Open Radical Cystectomy (ORC) in terms of major complications and positive surgical margins.^3^, ^4^, ^5^

RARC poses specific anesthetic challenges, including the need for deep neuromuscular blockade, precise fluid management, adjustments in pulmonary ventilation, hemodynamic control, and meticulous patient positioning. In contrast, ORC, while also complex, presents fewer anesthetic considerations and can be performed under regional anesthesia even in older patients.^6^, ^7^, ^8^, ^9^, ^10^

Anesthetic-surgical implications for ORC and RARC are still poorly described in the literature. Therefore, this study aimed to compare the incidence of transoperative complications in a university hospital over a five-year period, focused on anesthetic implications. We hypothesized that RARC would be associated with fewer transoperative complications than ORC.

## Methods

This retrospective comparative cohort study was conducted in a tertiary care university hospital by analyzing data from electronic medical records of patients who underwent ORC or RARC between March 2019 and March 2024. The project was approved by the Institutional Review Board, and research and methods adhered to the provisions of the Declaration of Helsinki and the STROBE guidelines.

Eligible participants were all adult patients (aged ≥ 18-years) undergoing radical cystectomy with an American Society of Anesthesiologists (ASA) physical status I, II, or III. Patients with ASA IV or V, patients undergoing emergency surgery, patients undergoing radical cystectomy exclusively under regional anesthesia (spinal + epidural), and those undergoing radical cystectomy combined with other surgical interventions were excluded from the study. Cases with incomplete information on critical variables were excluded in advance.

Radical cystectomy was indicated for muscle-invasive urothelial carcinoma of the bladder or non-muscle-invasive disease refractory to transurethral resection and/or intravesical therapy.

Both ORC and RARC used balanced general anesthesia with sevoflurane and remifentanil. In cases of open surgery, general anesthesia was combined with epidural anesthesia using morphine and ropivacaine. Continuous epidural analgesia was employed with intermittent boluses administered at the patient’s request. The epidural catheter was maintained for up to 48 hours postoperatively and removed based on clinical outcomes.

### Transoperative complications

For this study, transoperative complications were defined as anesthesia-related adverse events that occurred during or immediately after surgery. The following complications were analyzed:

• Arterial hypotension: defined as Mean Arterial Pressure (MAP) < 60 mmHg, sustained for more than one minute, based on continuous intraoperative monitoring;
• Need for vasoactive drugs;
• Ventilatory complications: hypoxemia – considered present when oxygen saturation as measured by pulse oxymetry (SpO_2_) was < 90%, sustained for more than five minutes during the procedure; and hypercarbia – defined as End-Tidal Carbon Dioxide (ETCO_2_) > 45 mmHg with continuous capnography monitoring, sustained for more than two minutes during the procedure;
• Neurological complications, such as cognitive dysfunction;
• Neurological or dermatological events attributable to patient positioning;
• Need for additional opioid administration in the immediate postoperative period (as documented in the medical records);
• In-hospital mortality (deaths during hospitalization).

### Outcomes

The following outcomes were assessed:

• Intraoperative outcomes – bleeding volume, need for blood transfusion, arterial hypotension, need for vasoactive drugs, ventilatory complications (hypoxemia and hypercarbia), and operating time;
• Postoperative outcomes – additional opioid requirements in the immediate postoperative period, neurological or dermatological injuries associated with patient positioning, neurological complications (e.g., cognitive dysfunction), major complications (Clavien-Dindo grade ≥ III), and in-hospital mortality;
• Recovery times – length of stay in the Intensive Care Unit (ICU), time to resume oral intake, and total length of hospital stay.

### Statistical analysis

The number of cases in the hospital during the study period determined the sample size. SPSS version 28.0 (IBM SPSS, Armonk, NY, USA) was used for statistical analysis. Descriptive statistics are presented as mean ± standard deviation or frequency and percentage. The Shapiro-Wilk test was used to assess the normality of data distribution. Continuous variables were compared using Student’s *t*-test or Mann-Whitney test, as appropriate. Categorical variables were analyzed using the Chi-Square test or Fisher’s exact test.

Ridge regression was employed to identify predictors of transoperative complications (defined as the presence of prolonged hypotension, need for vasopressors, or ventilatory complications). The following variables were included in the model: age, sex, body mass index, ASA classification, surgical approach (RARC vs. ORC), and operating time. Crude Odds Ratios (OR) were calculated to estimate the probability of outcome occurrence. Correlations between variables were also performed, with a correlation coefficient (*r*) of ≤ 0.39 considered weak, 0.40‒0.59 considered moderate, and ≥ 0.60 considered strong. To strengthen the internal validity of our between-group comparisons, we conducted a 1:1 propensity score matching, using age and ASA classification as predictors of complications.

As all assessed outcomes were essential information on critical variables, there were no missing data in our study. The significance level was set at p < 0.05 for all analyses.

## Results

Of 59 patients initially identified, 13 were excluded for undergoing radical cystectomy exclusively under regional anesthesia (spinal + epidural), and two were excluded for undergoing combined surgical procedures (one with a hysterectomy and another with a nephrectomy). Therefore, our sample consisted of 44 patients: 29 who underwent ORC, and 15 who underwent RARC. The groups did not differ significantly in age, sex, or ASA physical status, ensuring a homogeneous sample (Table 1).

**Table 1:** Sample characteristics.

Variable	Open surgery (n = 29)	Robotic surgery (n = 15)	p
Age (years), mean ± SD [95% CI]	68.2 ± 7.4 [65.4 – 71.0]	66.8 ± 6.9 [63.0 – 70.6]	0.512
Male, n (%) [95% CI]	24 (82.8) [24.7 – 27.5]	12 (80.0) [23.5 – 27.3]	0.752
BMI (kg.m^-2^), mean ± SD	26.1 ± 3.8	25.4 ± 3.5	0.601
ASA I‒II, n (%)	13 (44.8)	8 (53.3)	0.435
ASA III, n (%)	16 (55.2)	7 (46.7)	0.518

ASA, American Society of Anesthesiologists; BMI, Body Mass Index; CI, Confidence Interval; SD, Standard Deviation.

The most prevalent comorbidities were hypertension, diabetes mellitus, and chronic obstructive pulmonary disease (data not shown). Preoperative hemoglobin levels ranged from 9.9 to 15.1 g.dL^-1^.

The Ridge regression model showed that ASA III classification tended to be associated with a higher risk of complications (positive coefficient), but without statistical significance (p > 0.05). None of the other variables showed a significant association with anesthetic outcomes in the adjusted model. The discriminatory power of the model (Area Under the Curve [AUC]) was 0.64, indicating modest predictive ability.

Propensity score matching revealed statistically significant differences in the need for blood transfusion (p = 0.000004) and vasoactive drug use (p = 0.0003), both of which were significantly lower in the RARC group. In addition, there was a trend toward lower opioid use (p = 0.069) and hypotension (p = 0.082) in this group, without reaching statistical significance, however.

### Intraoperative outcomes

Intraoperative complications per group are shown in Table 2. There was no statistically significant difference between the groups in the incidence of hypotension (OR = 0.53, p = 0.492), vasoactive drug use (OR = 0.56, p = 0.512), or ventilatory complications (p > 0.05). Patients undergoing RARC experienced significantly less intraoperative bleeding (410.0 ± 185 mL) than those undergoing ORC (662.5 ± 210 mL) (p = 0.002). While the odds of requiring blood transfusion were lower in the RARC group (OR = 0.29), there was no significant difference in transfusion requirements between the groups (p = 0.110). The correlation between bleeding volume and need for transfusion was strong (*r* = 0.78; p < 0.001), whereas the correlation between blood loss and vasoactive drug use was weak (*r* = 0.25; p = 0.130).

**Table 2:** Intraoperative outcomes.

Variable	Open surgery (n = 29)	Robotic surgery (n = 15)	p
Bleeding volume (mL), mean ± SD [95% CI]	662.5 ± 210 [582.6 – 742.4]	410.0 ± 185 [307.6 – 512.4]	0.002
Need for blood transfusion, n (%)	13 (44.8)	3 (20.0)	0.110
Arterial hypotension, n (%)	14 (48.3)	6 (40.0)	0.492
Use of vasoactive drugs, n (%)	13 (44.8)	5 (33.3)	0.512
Hypoxemia, n (%)	2 (6.9)	1 (6.7)	0.980
Hypercarbia, n (%)	3 (10.3)	2 (13.3)	0.722
Operating time (min) mean ± SD [95% CI]	270 ± 50 [251.0 – 289.0]	340 ± 60 [306.8 – 373.2]	< 0.0001

CI, Confidence Interval; SD, Standard Deviation.

The data indicate a significantly longer operating time for RARC (340 ± 60 min) than for ORC (270 ± 50 min) (p < 0.0001).

### Postoperative outcomes

Postoperative complications per group are shown in Table 3. The data show a significantly higher need for additional postoperative opioid analgesia in the ORC group (OR = 0.12, p = 0.004). There was no statistically significant difference between the groups regarding the incidence of major complications (Clavien-Dindo grade ≥ III) (OR = 1.25, p = 0.742), or in-hospital mortality rates (24.1% in ORC vs. 20.0% in RARC; p = 1.00).

**Table 3:** Postoperative outcomes.

Variable	Open surgery (n = 29)	Robotic surgery (n = 15)	p
Additional opioid use^a^, n (%)	22 (75.9)	5 (33.3)	0.004
Clavien-Dindo grade ≥ III, n (%)	8 (27.6)	5 (33.3)	0.742
In-hospital mortality, n (%)	7 (24.1)	3 (20.0)	1.00

a Number of patients requiring at least one additional dose of opioid after the end of the surgical procedure.

Neither group experienced neurological or dermatological injury due to patient positioning. None of the patients exhibited any neurological or cognitive dysfunction that could be detected without specialized evaluation.

### Recovery times

Recovery times per group are shown in Table 4. There was no statistically significant difference between the groups in length of ICU stay (p = 0.351), time to resume oral intake (p = 0.352), or total length of hospital stay (p = 0.980). The correlation between operating time and total length of hospital stay was weak (*r* = 0.18; p < 0.21).

**Table 4:** Recovery times.

Variable	Open surgery (n = 29)	Robotic surgery (n = 15)	p
Length of ICU stay (days) mean ± SD [95% CI]	2.1 ± 1.8 [1.4 – 2.8]	1.9 ± 1.5 [1.1 – 2.7]	0.351
Time to resume oral intake (days) mean ± SD [95% CI]	3.8 ± 1.5 [3.2 – 4.4]	3.6 ± 1.4 [2.8 – 4.4]	0.352
Total length of hospital stay (days) mean ± SD [95% CI]	10.2 ± 4.1 [8.6 – 11.8]	10.1 ± 3.9 [7.9 – 12.3]	0.980

CI, Confidence Interval; ICU, Intensive Care Unit; SD, Standard Deviation.

## Discussion

Our results indicate that both surgical approaches are safe from an anesthetic perspective, ensuring hemodynamic stability and maintaining intraoperative ventilation. While the robotic approach resulted in less blood loss, potentially contributing to less hemodynamic instability, this did not significantly affect the need for blood transfusion. RARC also required less additional postoperative opioids.

Our findings are consistent with previous studies showing less blood loss in RARC, but with varying results regarding the need for transfusion.^3^^,^^11^ In a recent meta-analysis by Khetrapal et al., RARC was associated with a shorter length of hospital stay, less blood loss, fewer transfusions, and a lower incidence of thromboembolic events, although with longer operating time than ORC.^12^ Despite a lower incidence of blood transfusions in our study, this finding did not achieve statistical significance. Our data also align with the observation of longer operating time for RARC.^12^ At our center, we are in the early phase of the learning curve for RARC, which may have had an impact on operating times and, potentially, on complication rates.^13^ Additionally, more than one surgeon performed the procedures without controlling for individual experience, which could introduce variability in the results. However, it is worth noting that most of our reference studies also involved procedures performed by multiple surgeons.

Both open and robotic approaches used balanced general anesthesia, with open surgery incorporating epidural anesthesia. Nevertheless, the ORC group still required more additional postoperative opioids than the RARC group. This suggests that open surgery may be associated with a more intense pain response, even with epidural analgesia. This finding aligns with current literature indicating that the minimally invasive nature of robotic surgery results in less surgical trauma, which lessens the endocrine-metabolic response and, consequently, decreases the need for opioid analgesia, contributing to a more comfortable recovery with fewer opioid-related adverse effects.^14^^,^^15^

Our study found no hypoxemia or hypercarbia, suggesting that the ventilation strategies employed were sufficient to maintain adequate pulmonary ventilation in both surgical approaches. These findings align with those of Vejlgaard et al., who concluded that the prolonged Trendelenburg position combined with pneumoperitoneum, while challenging for anesthesia management, can be used safely in robotic surgery with appropriate ventilation adjustments.^16^ Specifically, Pressure-Controlled Ventilation (PCV) has demonstrated superior efficacy over volume-controlled ventilation in robotic and laparoscopic surgery. PCV has been shown to improve lung compliance and oxygenation in procedures performed with pneumoperitoneum, which may facilitate the adoption of protective tidal volumes and reduce the risk of barotrauma and atelectasis.^17^ We routinely use the PCV with Volume-Guarantee (PCV-VG) mode intraoperatively for patients in the head-down position.^18^

No significant difference was observed in intraoperative arterial hypotension between ORC and RARC. This is noteworthy because open surgery is typically associated with increased bleeding and involves neuraxial blockade, both of which can lower blood pressure. Our findings suggest that intraoperative bleeding was not sufficient to cause such changes, and that the sympathetic blockade was well-managed to maintain hemodynamic stability. These results indicate that arterial hypotension is more closely related to factors such as anesthetic management and individual patient hemodynamic response than to the surgical approach. While intraoperative hypotension can be linked to postoperative ischemic events, such as cerebral and myocardial ischemia, we found no documented cases of neurological or ischemic cardiac complications in our patients in the immediate postoperative period or during hospitalization. However, given the retrospective nature of our study, we did not systematically collect data from complementary tests (e.g., serial troponin measurements or neurological imaging) that would have detected these specific complications. Therefore, we lack sufficient data for a robust analysis on this matter and suggest that future prospective studies include more specific monitoring to address this issue.

Major complications and in-hospital mortality did not differ significantly between ORC and RARC. These findings align with previous research, including the RAZOR trial^3^ and Clement et al.’s meta-analysis.^4^ However, the mortality rate in both groups was high. This can be attributed to the complex patient profile at our institution. As a tertiary care university hospital, we predominantly treat patients with advanced disease and multiple comorbidities, often referred from other facilities after failure of initial therapy. This context differs substantially from centers that perform earlier interventions or treat lower-risk patients. Additionally, the still limited volume of cases per year at our center and our team’s early phase of the learning curve with the robotic approach may have negatively influenced the results, especially in the RARC group. It is also important to note that the study period (March 2019 to March 2024) includes the COVID-19 pandemic, during which surgical procedures were curtailed to prioritize pandemic-related burdens.

Our study found no significant difference in ICU or total hospital stay between ORC and RARC. This aligns with the systematic review by Rahman et al., who concluded that robotic surgery does not significantly reduce total length of stay.^19^ However, the literature presents conflicting results. Some studies suggest that robotic surgery, when combined with the Enhanced Recovery After Surgery (ERAS) protocol, can reduce hospital stay. The meta-analysis by Williams et al. showed that rigorous ERAS implementation reduced average hospital stay by up to 4.5 days, regardless of surgical or anesthetic technique.^20^ Conversely, Courboin et al. observed a significant reduction in hospital stay in the robotic surgery group, without any additional care.^21^ While we recognize the benefits of the ERAS protocol, it has not been systematically implemented at our institution. However, some of its components are routinely applied in our clinical practice, such as reduced fasting time, early oral liquid and solid re-feeding, early mobilization, multimodal analgesia with anesthetic blocks or wound infiltration, prevention of hypothermia, and opioid-sparing anesthesia.

Time to resume oral intake did not differ significantly between ORC and RARC. This suggests that gastrointestinal function recovery is similar regardless of the surgical technique.^3^^,^^11^^,^^22^ In contrast, a potential trend toward faster gastrointestinal function recovery has been observed in patients undergoing RARC.^23^ Additionally, alvimopan, an opioid receptor antagonist acting selectively in the intestine without compromising central opioid analgesia, significantly reduces time to first bowel movement, especially when combined with the ERAS protocol.^24^ Unfortunately, alvimopan is not currently available in Brazil.

While Ridge logistic regression helped address collinearity, the small number of events and limited sample size, particularly in the RARC group, compromised the statistical robustness of the model. The predictive ability was modest (AUC = 0.64), and no variables reached clear statistical significance. ASA III classification hinted at a possible association with increased complication risk. Propensity scores matching results suggest that the hemodynamic benefits of the robotic approach may persist even after controlling for important clinical confounders, supporting its potential as a less invasive and physiologically stable technique compared with the open approach. We recommend that future studies with larger sample sizes explore more robust predictive models to further investigate these associations.

Our study has limitations. First, all data included in the study were collected from electronic hospital records, and the variables used in our analysis were mandatory fields (e.g., age, sex, ASA, duration of surgery, anesthetic events). To ensure the integrity of our database, we performed a complementary manual check during data extraction, and cases with incomplete information on critical variables were excluded. However, we acknowledge that this methodology may introduce selection bias. Second, as a single-center study, the results may not be directly generalizable to settings with different characteristics. Third, a formal sample size calculation was not performed due to the exploratory and retrospective design of our study, which may have led to the study being underpowered to detect significant differences, particularly for low-incidence outcomes. Confidence intervals and clinical relevance should guide data interpretation, reinforcing the need for prospective studies with larger sample sizes. Fourth, the only statistically significant differences between groups were for intraoperative blood loss and opioid use, which were not adjusted for potential confounders such as baseline hemoglobin, institutional analgesic protocol, or individual anesthetic technique. Due to the retrospective nature and small sample size of our study, robust multivariate adjustments would have compromised statistical validity, especially in the RARC group. However, we noticed similar methodological limitations in previous studies we consulted, which also did not adjust for these specific variables. Further prospective studies with greater control over baseline clinical variables are needed to better address these issues. Fifth, we analyzed anesthetic complications individually to preserve the clinical specificity of each outcome, given the diverse pathophysiological mechanisms and multiple clinical implications associated with each event. We did not define a hierarchy of severity or a composite outcome because the frequency and clinical impact of these variables differ substantially. We recognize, however, that the lack of a consolidated index may limit integrated comparisons between groups. Future investigations may benefit from the development of clinically weighted severity scores or composite outcomes. Additionally, the administration of vasoactive drugs does not depend solely on the intensity of intraoperative hypotension. It is a multifactorial clinical decision influenced by several factors, including the patient’s baseline hemodynamic status, existing cardiovascular comorbidities, response to fluid replacement, institutional protocols, and the anesthesiologist’s preferences. Therefore, while the need for amines was analyzed as a marker of hemodynamic instability, the specificities of this indication warrant a separate analysis. Finally, we did not differentiate between anesthetic techniques; however, our focus was on evaluating transoperative complications within the broader surgical context (surgery and anesthesia combined), rather than comparing specific anesthetic techniques.

## Conclusion

While RARC was associated with reduced blood loss and lower additional postoperative opioid use, our small retrospective sample did not identify significant differences in intraoperative hemodynamic parameters or major complications. The surgical technique had no impact on in-hospital mortality. Further prospective controlled studies are needed to confirm these findings.

## Ethical approval

The project was approved by the Institutional Review Board of Hospital Universitário Pedro Ernesto of Universidade do Estado do Rio de Janeiro (HUPE-UERJ) (approval n° 6.600.371), and all research and methods adhered to the provisions of the Declaration of Helsinki and the STROBE guidelines.

## Data availability

The data that support the findings of this study are available from the corresponding author, SLLM, upon reasonable request.

## Authors’ contribution

Sérgio Luiz do Logar Mattos: Conception and design of the study, analysis and interpretation of data; Drafting the article.

Ronaldo Damião: Conception and design of the study; revising the article critically for important intellectual content.

Fabrício Borges Carrerette: Conception and design of the study; analysis and interpretation of data; drafting the article.

Aretha Paes de Lima Carneiro: Acquisition of data; analysis and interpretation of data; drafting the article.

Ian Maia Fontes: Acquisition of data; drafting the article.

## Funding

This research did not receive any specific grant from funding agencies in the public, commercial, or not-for-profit sectors.

## Conflicts of interest

The authors declare no conflicts of interest.
